# Strong relationship between cholesterol, low-density lipoprotein receptor, Na^+^/H^+^ exchanger, and SARS-COV-2: this association may be the cause of death in the patient with COVID-19

**DOI:** 10.1186/s12944-021-01607-5

**Published:** 2021-12-13

**Authors:** Erkan Cure, Medine Cumhur Cure

**Affiliations:** 1 Department of Internal Medicine, Bagcilar Medilife Hospital, 34200 Istanbul, Turkey; 2 Department of Biochemistry, Private Kucukcekmece Hospital, Istanbul, Turkey

**Keywords:** Cholesterol, Lipids, Na^+^/H^+^ exchanger, PCSK9, COVID-19, SARS-CoV-2

## Abstract

Lipids have a wide variety and vital functions. Lipids play roles in energy metabolism, intracellular and extracellular signal traffic, and transport of fat-soluble vitamins. Also, they form the structure of the cell membrane. SARS-CoV-2 interacts with lipids since its genetic material contains lipid-enveloped ribonucleic acid (RNA). Previous studies have shown that total cholesterol, high-density lipoprotein, and low-density lipoprotein (LDL) levels are lower in patients with severe novel coronavirus disease 2019 (COVID-19) compared to patients with non-severe COVID-19.

Na^+^/H^+^ Exchanger (NHE) is an important antiport that keeps the intracellular pH value within physiological limits. When the intracellular pH falls, NHE is activated and pumps H^+^ ions outward. However, prolonged NHE activation causes cell damage and atherosclerosis. Prolonged NHE activation may increase susceptibility to SARS-CoV-2 infection and severity of COVID-19.

In COVID-19, increased angiotensin II (Ang II) due to angiotensin-converting enzyme-2 (ACE2) dysfunction stimulates NHE. Lipids are in close association with the NHE pump. Prolonged NHE activity increases the influx of H^+^ ions and free fatty acid (FFA) inward. Ang II also causes increased low-density lipoprotein receptor (LDLR) levels by inhibiting proprotein convertase subtilisin/kexin type 9 (PCSK9). Thus, intracellular atheroma plaque formation is accelerated.

Besides, SARS-CoV-2 may replicate more rapidly as intracellular cholesterol increases. SARS-CoV-2 swiftly infects the cell whose intracellular pH decreases with NHE activation and FFA movement. Novel treatment regimens based on NHE and lipids should be explored for the treatment of COVID-19.

## Introduction

Lipids have a wide variety and crucial functions. Lipids play roles in energy metabolism, intracellular and extracellular signal traffic, and transport of fat-soluble vitamins. Also, they form the structure of the cell membrane [1]. Lipids are used as building blocks and energy sources in the membranes of viral organelles formation, so lipids are vital during viral infection [1]. The regulation of macrophages and many immunomodulatory pathways are associated with lipids, and lipids are involved in pulmonary infection diseases and inflammatory conditions [2]. Ribonucleic acid (RNA) viruses require some particles for viral entry into host cells and to escape the host immune system. Lipids are needed for intercellular and intracellular signaling during the production of particles required for RNA viruses [2]. SARS-CoV-2 interacts with lipids since its genetic material contains lipid-enveloped RNA [3]. Previous studies have shown that total cholesterol, high-density lipoprotein, and low-density lipoprotein (LDL) levels are lower in patients with severe novel coronavirus disease 2019 (COVID-19) compared to patients with non-severe COVID-19 [4].

Na^+^/H^+^ exchanger (NHE) is a crucial antiport that keeps the intracellular pH value within certain limits. NHE pumps H^+^ ions outward when the intracellular pH drops. Recently, it has been suggested that NHE may increase susceptibility to SARS-CoV-2 and the severity of COVID-19 [5, 6]. Interestingly, there is also an interaction between lipids and NHE [7]. Here, we examined whether there is a triple interaction between SARS-CoV-2, lipids, and NHE.

## SARS-CoV-2 and cholesterol

Cholesterols act as a conductor during the entry of SARS-CoV-2 into the cell by fusing with angiotensin-converting enzyme-2 (ACE2); therefore, high cholesterol levels may increase susceptibility to SARS-CoV-2 [8]. According to this theory, lowering cholesterol with statin therapy can reduce viral replication. On the other hand, cholesterol plays a vital role in the gathering, replication, and infectivity of the viral RNA of SARS-CoV-2 [9]. A previous study reported that cholesterol metabolism-related proteins such as LDL, LDL receptor (LDLR)-related protein (LRP), Apo B, Apo E were diminished in SARS-CoV-2 infected human colon epithelial carcinoma cells [10]. In most studies investigating lipid levels in patients with COVID-19, it has been shown that low lipid levels increase mortality and disease severity in COVID-19 [11]. The virus can interact intensively with cholesterol, causing it to shift into the cell, and it may use cholesterol, lowering its level.

On the other hand, LDL carries a large percentage of plasma Coenzyme Q10 (CoQ10), which has a significant antioxidant capacity and prevents peroxidative damage to cellular membranes [12]. Low LDL levels may contribute to endothelial dysfunction by causing a decrease in plasma CoQ10 levels. As a result, low serum cholesterol levels may increase the severity and damage of COVID-19.

## Lipid raft and SARS-CoV-2

Lipid rafts are dynamic regions in the cell membrane ranging from 10 to 200 nm. They contain sphingolipids, glycosphingolipids, cholesterol, and glycosylphosphatidylinositol-associated proteins [13]. Lipid rafts are involved in tasks like intercellular signal transduction, trafficking, endocytosis, and viral infection [14]. Cholesterol is an essential component of lipid rafts and interferes with various aspects of the virus life cycle, particularly viral entry [15, 16]. Coronavirus and other many enveloped viruses enter cells through cholesterol in viral and cellular membranes [17]. SARS-CoV-2 associates with ACE2 in lipid rafts and starts a signaling cascade that allows virus endocytosis [2, 14]. That is, SARS-CoV-2 enters the cell using lipid rafts.

## Statins, Proprotein convertase Subtilisin/Kexin type 9 (PCSK9) inhibitors and COVID-19

Statins have anti-inflammatory, antioxidant, and anti-thrombotic effects [18]. They also lead to ACE2 upregulation in heart and kidney tissue [18]. By lowering cholesterol, statins and PCSK9 inhibitors can block entry of SARS-CoV-2 into the host cell or inhibit its replication [19]. If low cholesterol levels are associated with COVID-19 mortality, then is lowering cholesterol levels the eligible treatment? Detailed studies are needed on this subject.

On the other hand, statins may exert a direct antiviral effect by interacting with the main protease enzyme of SARS-CoV-2 [20]. PCSK9 inhibitors may exert potent antiviral effects against SARS-CoV-2 by increasing type I interferon levels [21].

## SARS-CoV-2 and LDLR

LDLR is the structure within the cell containing the type I transmembrane protein family that removes cholesterol-carrying lipoproteins from the plasma circulation [22]. Viruses that settle inside the cell enter the cell through cholesterol, interact with LDLR and cause infection [23]. LRP-1 is a protein belonging to the LDLR family. Human cytomegalovirus can easily fuse with the plasma membrane by increasing the expression of LRP-1 [24]. When the intracellular pH is acidic, the glycoprotein of the vesicular stomatitis virus interacts with the LDLR-domain, thus the virus causes the infection [25]. Some serotypes of human rhinoviruses also enter cells through this family of receptors [26]. Hepatitis C virus increases LDLR expression while suppressing PCSK9 expression. Thus, it effortlessly infects the cell [27]. Same way as the viruses mentioned above, SARS-CoV-2 can also interact with LDLR while using cholesterol for RNA replication. As a result of this interaction, intracellular cholesterol storage may increase. HMG-CoA reductase activity and cholesterol synthesis decrease when intracellular cholesterol storage increases [28]. Therefore, patients with COVID-19 may have low plasma cholesterol, which may be evidence that the virus affects LDLR.

## SARS-CoV-2, angiotensin (Ang) II, and LDLR mediated plaque formation

SARS-CoV-2 causes infection by fusing with ACE2. ACE2 converts Ang II to Ang 1–7 and 9des-bradykinin to its metabolites [29]. Ang II causes vasoconstriction and increased oxidative stress, while Ang 1–7 has vasodilator and antioxidant effects [5]. When the virus fuses with ACE2, Ang II degradation stops, and its level increases [29]. Ang II is directly related to lipid metabolism as well as its oxidant effects. Ang II decreases PCSK9 expression in aortic smooth muscle cells and thus increases the number of LDLRs in the membrane [30]. When the number of LDLR increases, lipid accumulation occurs in the cell membrane and within the cell, and the lipid level in the blood decreases [31]. Although this effect seems beneficial, Sendra et al. described a mechanism by which Ang II causes cholesterol infiltration in the arteria intima by upregulating LRP1 [32]. Most COVID-19 patients with mortality have obesity, diabetes, hypertension, and atherosclerotic heart disease. These patients are already predisposed to atherosclerosis, and excessive Ang II levels during COVID-19 may upregulate LDLR, accelerating intimal plaque formation. Since the increase in Ang II also increases the tendency to thrombosis, patients with COVID-19 may die suddenly from a heart attack even though their blood cholesterol is low.

Additionally, acute phase response during COVID-19 may lead to oxide-LDL formation and LDLR upregulation [12]. Oxide-LDL via lectin-like oxidized LDLR-1 (LOX-1) activation leads to ACE gene expression [33]. Upregulation of ACE downregulates ACE2, vice versa [29]. Ang II level may be excessively elevated in patients with COVID-19 since ACE2, which has already fused with the virus, is also dysfunctional. Therefore, Ang II-LDLR-related endothelial dysfunction and arterial plaque formation may be excessively accelerated.

## Relationship between lipids, NHE and COVID-19

When the intracellular pH drops, NHE is physiologically activated. NHE pumps 3Na^+^ ions into the cell and 2H^+^ out of the cell. NHE rapidly brings the intracellular pH to a physiological level, and its activity ceases [5]. There is a close relationship between NHE and lipids. Megalin is a member of the LDLR family, which is abundant in proximal tubular cells. Also, it is found in type II pneumocyte, brain, epididymis, eye, ear, and thyroid tissues [34]. The virus may infect all tissues containing megalin. Previous work has shown that megalin interacts with NHE3 through the NHE regulatory cofactor. In this way, there is a connection between NHE3 and megalin [34]. This interaction is also likely to occur in other tissues containing megalin. There is a strong interaction between Angiotensinogen, Ang II, and megalin. This interaction accelerates the atherosclerotic process [35]. Since Ang II is the potent activator of NHE, megalin also stimulates NHE indirectly.

Besides, lipid raft affects NHE1 and NHE3 activity [7, 36]. Intestinal NHE3 activity and Na^+^ absorption increased in the postprandial period [36]. A previous study showed that increased NHE activity in the liver tissue of rats increased the extracellular H^+^ ion flux. At the end of this event, the transmembrane movement of free fatty acids (FFA) increases and FFA and H^+^ ions pass into the cell [37]. The influx of H^+^ ions with the FFA into the cell lowers the intracellular pH and activates NHE again [37]. This process can become a vicious circle in the patient with COVID-19.

On the other hand, in tissues such as the pancreas and adipose tissue, the movement of FFA into the cell acidifies the intracellular pH [38, 39]. Insulin promotes the conversion of FFA to triglycerides and activates NHE [5, 39]. During this event, the intracellular pH increases due to the outflow of H^+^ ions from the intracellular space. Insulin deficiency or insulin resistance causes an increase in FFA flow into the cell and a decrease in intracellular pH [39]. Intracellular FFA efflux causes inhibition of glycolysis and glucose intolerance [38]. Ang II causes insulin resistance by increasing oxidative stress [40], and patients with COVID-19 have insulin resistance due to the increase in Ang II [41]. As a result of these events, intracellular FFA influx and prolonged NHE activation may accelerate the atherosclerotic process in patients with COVID-19.

SARS-CoV-2 main protease activity is easily affected by intracellular pH, and its infectivity increases at low pH values [42]. Therefore, increasing the intracellular pH has been one of the cornerstones of the treatment of COVID-19. Interestingly, in patients with obesity, diabetes, hypertension, and cardiac disease, the intracellular pH is acidic compared to normal individuals [5]. Ang II level is high in these patients, and there is a prolonged NHE stimulation. During prolonged NHE activation, H^+^ ions are pumped out of the cell. As a result of prolonged NHE pumping, reactive oxygen radicals increase outside the cell. With the redox reaction, H^+^ ions are returned to the cell. Thus, the intracellular pH becomes acidic [5]. The interaction between NHE and FFA contributes to intracellular pH reduction by an influx of FFA and H^+^ into the cell. SARS-CoV-2 can decrease intracellular pH by interacting with lipids and NHE either directly or indirectly by increasing Ang II. Thus, at acidic intracellular pH, the virus can easily invade the cell. On the other hand, extracellular H^+^ increase and intracellular lipid accumulation can cause accelerated atherosclerosis and sudden heart attack [43, 44].

Physiologically, PCSK9 activity increases as the intracellular pH becomes acidic [45]. PCSK9 binds to and breaks down LDLR in liver cells. LDLR, which cannot return to the cell surface, cannot enter the liver cells, and its level increases within the cell [46]. Interestingly, SARS-CoV-2 may suppress PCSK9 by increasing Ang II and increased LDLR levels. At acidic intracellular pH, LDL dissociates from LDLR. LDLR returns to the cell surface. LDL separated from its receptor is broken down into cholesterols in lysosomes. The released cholesterol is used by the cell [47]. SARS-CoV-2 may use the increased cholesterol within the cells for replication and signal transduction. Prolonged NHE activation and intracellular lipid accumulation can increase the replication of the virus and cause it to spread effortlessly.

Another paramount issue is that LDL reduces platelet functions by inhibiting NHE activation in platelets, thus exerting an antithrombotic effect [48]. However, this effect is only seen in native LDL at a physiological level. In SARS-CoV-2 infection, while native LDL decreases, the oxidized LDL levels increases [49]. In addition to other mechanisms, a decrease in LDL levels in patients with COVID-19 may also cause an increase in thrombotic events.

## Conclusion

ACE2 blockade in COVID-19 increases Ang II, leading to prolonged NHE activity. The activity acidifies the intracellular pH with redox reactions. FFAs move into the cell with H^+^ ions. High cholesterol inside the cell can accelerate atherosclerosis and cause sudden cardiac death. Besides, SARS-CoV-2 may replicate more rapidly as intracellular cholesterol increases. SARS-CoV-2 straightforwardly infects the cell whose intracellular pH decreases with NHE activation and FFA movement. Novel treatment regimens based on NHE and lipids should be explored for the treatment of COVID-19.

## Data Availability

Not applicable.
